# Changes in Thalamic Connectivity in the Early and Late Stages of Amnestic Mild Cognitive Impairment: A Resting-State Functional Magnetic Resonance Study from ADNI

**DOI:** 10.1371/journal.pone.0115573

**Published:** 2015-02-13

**Authors:** Suping Cai, Liyu Huang, Jia Zou, Longlong Jing, Buzhong Zhai, Gongjun Ji, Karen M. von Deneen, Junchan Ren, Aifeng Ren, for the Alzheimer’s Disease Neuroimaging Initiative

**Affiliations:** 1 School of Life Sciences and Technology, Xidian University, Xi’an, 710071, China; 2 Zhejiang Key Laboratory for Research in Assessment of Cognitive Impairments, Hangzhou, 311121, China; University of Pennsylvania, UNITED STATES

## Abstract

We used resting-state functional magnetic resonance imaging (fMRI) to investigate changes in the thalamus functional connectivity in early and late stages of amnestic mild cognitive impairment. Data of 25 late stages of amnestic mild cognitive impairment (LMCI) patients, 30 early stages of amnestic mild cognitive impairment (EMCI) patients and 30 well-matched healthy controls (HC) were analyzed from the Alzheimer’s disease Neuroimaging Initiative (ADNI). We focused on the correlation between low frequency fMRI signal fluctuations in the thalamus and those in all other brain regions. Compared to healthy controls, we found functional connectivity between the left/right thalamus and a set of brain areas was decreased in LMCI and/or EMCI including right fusiform gyrus (FG), left and right superior temporal gyrus, left medial frontal gyrus extending into supplementary motor area, right insula, left middle temporal gyrus (MTG) extending into middle occipital gyrus (MOG). We also observed increased functional connectivity between the left/right thalamus and several regions in LMCI and/or EMCI including left FG, right MOG, left and right precuneus, right MTG and left inferior temporal gyrus. In the direct comparison between the LMCI and EMCI groups, we obtained several brain regions showed thalamus-seeded functional connectivity differences such as the precentral gyrus, hippocampus, FG and MTG. Briefly, these brain regions mentioned above were mainly located in the thalamo-related networks including thalamo-hippocampus, thalamo-temporal, thalamo-visual, and thalamo-default mode network. The decreased functional connectivity of the thalamus might suggest reduced functional integrity of thalamo-related networks and increased functional connectivity indicated that aMCI patients could use additional brain resources to compensate for the loss of cognitive function. Our study provided a new sight to understand the two important states of aMCI and revealed resting-state fMRI is an appropriate method for exploring pathophysiological changes in aMCI.

## Introduction

Mild cognitive impairment (MCI) patients have a high rate of progressing to Alzheimer’s disease (AD), which is about 10–15% annually [1]. The subtypes of MCI based on the pattern of neuropsychological impairment have been proposed previously, where such distinction may mark incipient forms of different dementias [2]. With the advent of disease-modifying treatments for AD, the amnestic subtype of MCI (aMCI) in particular has received much attention as a possible precursor to this most common dementia state [3]. It has been reported that aMCI patients developed AD at a rate of 16% per year [4].

The early state of aMCI (EMCI) is characterized by patients showing minute memory impairment or other cognitive dysfunctions on neuropsychological tests and not completely meeting the diagnostic criteria of aMCI. However, compared to well-matched healthy controls (HC), EMCI subjects showed much difference between intelligence and memory scores or tests (e.g., EMCI subjects have MMSE scores between 24 and 28 vs. HC between 27 and 30). Norio et al. [5] made a comparison of the differences among EMCI, aMCI and HC using an 18F-fluorodeoxyglucose positron emission tomography (18F-FDG PET) examination and observed that EMCI had important significance in diagnosing the early-state of aMCI. As the late stage of aMCI, LMCI patients showed worse performance in most testing domains (e.g., LMCI subjects have MMSE scores between 20 and 26), possibly with a higher degree of medial temporal lobe atrophy (MTA), and being at the greatest risk of conversion to dementia. However, brief cognitive tests such as the MMSE are often subjective and insensitive to early-stage dementia, with objective evidence in distinguishing the LMCI and EMCI needing to be explored.

Recently, functional magnetic resonance imaging (fMRI) is increasingly being used to study the pathogenesis of neurodegenerative disorders. In particular, resting-state fMRI has attracted much more attention and has been widely used to investigate the pathogenesis of AD and MCI. Likewise, functional connectivity methods based upon low frequency (0.01–0.08Hz) spontaneous BOLD fluctuations in resting fMRI provide a powerful tool to characterize intrinsically functional associations among brain regions [6]. According to previous research, the thalamus is a crucial brain area which processes and integrates neural activity from widespread neocortical inputs and outputs [7] and is believed to coordinate information and facilitate communication (e.g., memory, attention, and perception) in a number of areas of the cerebral cortex [8, 9], which makes it extraordinarily interesting in the study of functional connectivity between the thalamus and other functional brain regions. Zhang et al. [10] calculated the correlations between the thalamus and cerebral cortex in adult human brains and highlighted the potential of resting-state fMRI imaging to elucidate thalamocortical relationships. Using independent component analysis (ICA) on resting-state fMRI data, Kim et al. [11] found that different functional subdivisions of the thalamus showed different functional network (There is great interest in defining brain “networks” from fMRI data. This is often attempted by identifying a group of two or more functional “nodes” such as spatial ROIs or ICA maps. The two or more functional “nodes” linked together to complete the corresponding brain function, hence the two or more functional “nodes” compose a brain network) connectivities between hemispheres.

What’s more, some studies have demonstrated that the thalamus is subjected to prominent volume loss and microstructural change with increasing age [12, 13]. Changes in thalamo-cortical connectivity may cause the decline in cognitive ability related to aging [14, 15]. Zarei [16] explored the pattern of thalamic degeneration in AD by combining diffusion tensor imaging (DTI) and structural magnetic resonance imaging (MRI), successfully identifying regional thalamic atrophy in AD and demonstrating that these regions are mainly connected with the hippocampus, temporal cortex, and prefrontal cortex. Some of these connections such as the connectivity of the thalamo-hippocampus are thought to be significant pathways for memory [17], and memory dysfunction is often the earliest and most remarkable symptom in AD and MCI patients [1, 18]. Using the method of DTI, Damoiseaux et al. [19] observed an impaired connection between the thalamus and the medial temporal cortex in AD patients. It is worth noting that connectivity between the thalamus and the temporal lobe is related to working/short-term memory [20, 21]. Recently, Wang et al [22] found that impairment and compensation of thalamus connectivity coexisted in MCI patients. Zhou and colleagues [23] deepened the research of thalamic functional connectivity and revealed disease severity-related alterations of the thalamo-cortical network in AD and MCI patients. However, as the two important subsets of aMCI, few studies have shown the connectivity pattern of the left and right thalamus in EMCI and LMCI. In the present work, we would like to further the study of the thalamus and we hypothesized that: (a) the thalamic connectivity with a set of brain regions would be abnormal in EMCI and/or LMCI patients; (b) functional connectivity pattern of the thalamus in the different state of aMCI patients would be different.

## Materials and Methods

### 1. Overview of ADNI

Data used in the preparation of this article were obtained from the Alzheimer’s disease Neuroimaging Initiative (ADNI) database (adni.loni.usc.edu). ADNI was launched in 2003 by the National Institute on Aging (NIA), the National Institute of Biomedical Imaging and Bioengineering (NIBIB), the Food and Drug Administration (FDA), private pharmaceutical companies and non-profit organizations, as a $60 million, 5-year public-private partnership. The primary goal of ADNI has been to test whether serial magnetic resonance imaging (MRI), positron emission tomography (PET), other biological markers, and clinical and neuropsychological assessment can be combined to measure the progression of mild cognitive impairment (MCI) and early Alzheimer’s disease (AD). Determination of sensitive and specific markers of very early AD progression is intended to aid researchers and clinicians in developing new treatments and monitor their effectiveness, as well as lessen the time and cost of clinical trials. For up-to-date information, see www.adni-info.org.

### 2. Subjects

A total of 96 subjects (31 LMCI; 33 EMCI; 32 HC) participated in the study. EMCI subjects had MMSE scores between 24–30 (inclusive) and LMCI subjects had MMSE scores between 20–26 (inclusive); a memory complaint had objective memory loss measured by education adjusted scores on the Wechsler Memory Scale Logical Memory II, a CDR of 0.5, absence of significant levels of impairment in other cognitive domains, essentially preserved activities of daily living, and an absence of dementia [24]. The functional brain MRI data and corresponding clinical data from baseline and follow-up scans were downloaded before November 24, 2013 from the ADNI publically available database. Data from 11 subjects (6 LMCI; 3 EMCI; 2 HC) were excluded due to excessive motion (see data preprocessing). Table 1 shows the details of clinical and demographic data for the remaining 85 subjects. No significant differences in gender or age were noted.

**Table 1:** Demographics and clinical information.

	LMCI(n = 25)	EMCI(n = 30)	HC(n = 30)	*P* value
Gender (M/F)	17/8	12/18	13/17	0.84 ^a^
Age (years)	74.63±7.11	72.13±8.1	75.8±7.14	0.16 ^b^
MMSE score	25.94±1.89	27.85±1.57	29.63±1.52	< 0.0001^b^
CDR score	1.39±0.21	0.51±0.13	0.00±0.00	< 0.0001^b^

Data are given as mean ± standard deviation (SD); MMSE, Mini-Mental State Examination; CDR, Clinical Dementia Rating

a: The *P* value was obtained by an independence Pearson chi-square test.

b: The *P* value was obtained by a one-way analysis of variance test.

### 3. Data Acquisition

All subjects were scanned on a 3.0-Tesla Philips MRI scanner. Resting-state functional images were obtained by using an echo-planar imaging sequence (EPI：a fast magnetic resonance imaging technique that allows acquisition of single images in as little as 20 msec and performance of multiple-image studies in as little as 20 seconds；for more information see [25]) with the following parameters: 140 time points; repetition time (TR) = 3000 ms; echo time (TE) = 30 ms; flip angle = 80°, number of slices = 48; slice thickness = 3.3 mm spatial resolution = 3×3×3 mm^3^ and matrix = 64×64. All original image files are available to the general scientific community.

### 4. Imaging Preprocessing

Data preprocessing was carried out using the Data Processing Assistant for Resting-State fMRI (DPARSF, Yan and Zang [26]; http://rfmri.org/DPARSF), which is based on Statistical Parametric Mapping software (SPM8) (http://www.fil.ion.ucl.ac.uk/spm/) and Resting-State fMRI Data Analysis Toolkit (REST; Song et al., [27]; http://restfmri.net). The first ten image volumes of resting-state data were discarded for the signal equilibrium and subjects’ adaptation to the fMRI scanning noise. The remaining 130 images were corrected for the timing differences between each slice and motion effects (six-parameter rigid body). Datasets with more than 1.5 mm maximum displacement in any of the x, y, or z directions or 1.5° of any angular motion were discarded, hence a total of 11 subjects (6 LMCI; 3 EMCI; 2 HC) were excluded. Next, we spatially normalized images to the standard EPI template (a built-in functional template in SPM, some of the normalized maps in the S1 Fig.), based on the Montreal Neurological Institute (MNI) stereotactic space, and then resampled them into 3mm×3mm×3 mm cubic voxels. The functional images were spatially smoothed with a Gaussian kernel of 6×6×6 mm^3^ full width at half maximum (FWHW) to decrease spatial noise. Following this, we removed the linear trends and temporally filter (0.01Hz < f < 0.08Hz). To remove any residual effects of motion and other non-neuronal factors, a Friston 24-parameter (6 head motion parameters, 6 head motion parameters at one time point before, and the 12 corresponding squared items) [28], as well as parameters for the white matter signal, global mean signal, and cerebrospinal fluid signal were used as nuisance variables in the functional connectivity analysis.

Recently, it has been reported that small head movement may have impact on certain resting-state fMRI metrics, such as the method of functional connectivity [29–32]. Therefore, we respectively evaluated the correlation between framewise displacement (FD) in accordance with the criteria of Power et al.[30] and the time series of the left/right thalami to check the impact of the head movement on the functional analysis of the thalamus, and then the correlation coefficients were converted to z values using Fisher’s r-to-z transform to improve normality. The results showed that there was no correlation between FD and time series of left/right thalami in each group (hypothesis: there is no correlation between FD and time series of left/right thalami, all of the *P* values were more than 0.05 (confidence interval: 95%)). For more details see Table 2.

**Table 2:** The correlation between framewise displacement (FD) and time series of left/right thalami in the three groups.

	LMCI_L	LMCI_R	EMCI_L	EMCI_R	HC_L	HC_R
***r***(m±SD)	.0014±.0720	.0400±.0634	.0258±.0868	.0266±0.1193	.0036±.0601	.0036±.0492
***P***	0.9240^c^	0.6570^c^	0.1200^c^	0.2400^c^	0.7560^c^	0.7060^c^
***t***	0.0960	0.4490	-1.6030	-1.2010	-0.3140	-0.3810

The correlation coefficient *r* is given as mean ± standard deviation (m±SD).

LMCI_L/R: The correlation between FD and time series of the left/right thalami in LMCI; EMCI_L/R: The correlation between FD and time series of the left/right thalami in EMCI; NC_L/R: The correlation between FD and time series of the left/right thalami in NC;

c: The *P* value was obtained by a one sample *t*-test (confidence interval: 95%), hypothesis: there is no correlation between FD and time series of bilateral thalami.

### 5. Definition of Seed Regions

The left and right thalamus ROIs were generated using an automated anatomic labeling (AAL) [33] template implemented with the Resting-State fMRI Data Analysis Toolkit V1.6 (REST, http://restfmri.net/forum/). We extracted the left and right thalami with the corresponding AAL threshold value (left thalamus: 77, right thalamus: 78). Each ROI was resampled to the spatial resolution of fMRI images using the 0 interpolation approach and then binarized to avoid a non-zero value.

### 6. Functional Connectivity Analysis

For each subject and the left/right thalamus ROIs, the BOLD time series of the voxels within the ROIs were averaged to generate the reference time series. Correlation analysis was carried out between the reference time series and the time series of all other brain voxels in the whole brain. Correlation coefficients were converted to z values using Fisher’s r-to-z transform to improve the normality [34].

### 7. Statistical Analysis

**7.1 Within-group functional connectivity analysis.** The individual z value was entered into a random effect one-sample *t*-test in a voxel-wise manner to determine brain regions showing significant connectivity to the left and right thalamus within each group. Within experimental groups a single voxel threshold was set at *P* < 0.05 and a minimum cluster size of 40 voxels was used to correct for multiple comparisons. This yielded a corrected threshold of *P* < 0.01 in each group, as determined by the Monte Carlo simulation (see program AlphaSim by D. Ward in AFNI software. Parameters were: single voxel *P* value = 0.05, FWHM = 6 mm with BrainMask.)

**7.2 Between-group functional connectivity analysis.** A one-factor analysis of variance (ANOVA) with age as covariance was performed at each voxel to determine the differences between the LMCI, EMCI and HC. The statistical maps were then set using *P* < 0.05 for each voxel and a cluster size of at least 40 voxels resulting in a corrected threshold of *P* < 0.01 based on Monte Carlo simulations. Subsequently, all the functional connectivity values were used for a post hoc analysis. Statistical comparisons of the functional connectivity values between each pair of the three groups were performed using a two-sample *t*-test with age as covariance.

### 8. Revealing the Detailed Condition of Decreased and Increased for the Identified Regions

“Decreased functional connectivity” could be due to 1) decrease in positive functional connectivity (decrease in absolute value); 2) decrease in negative functional connectivity (increase in absolute value); and 3) turn from positive functional connectivity into negative functional connectivity. The same situation applies to “increased functional connectivity. To carefully reveal the condition for the regions we identified, we extracted the correlation coefficients, and then the correlation coefficients were converted to z values by using Fisher’s r-to-z transform to improve the normality. Following this step, we did a statistical analysis by using a one sample *t*-test.

### 9. Relationship between Functional Connectivity Strength and Clinical Variables

Correlation analyses between functional connectivity strength (z scores) and the clinical variables (MMSE) were performed to investigate whether the functional connectivity strength had a relationship with the clinical variables. Because these analyses were exploratory, we used a statistical significance level of *p* < 0.05 (uncorrected).

## Results

### 1. Within-group Functional Connectivity Maps

**Fig 1:**
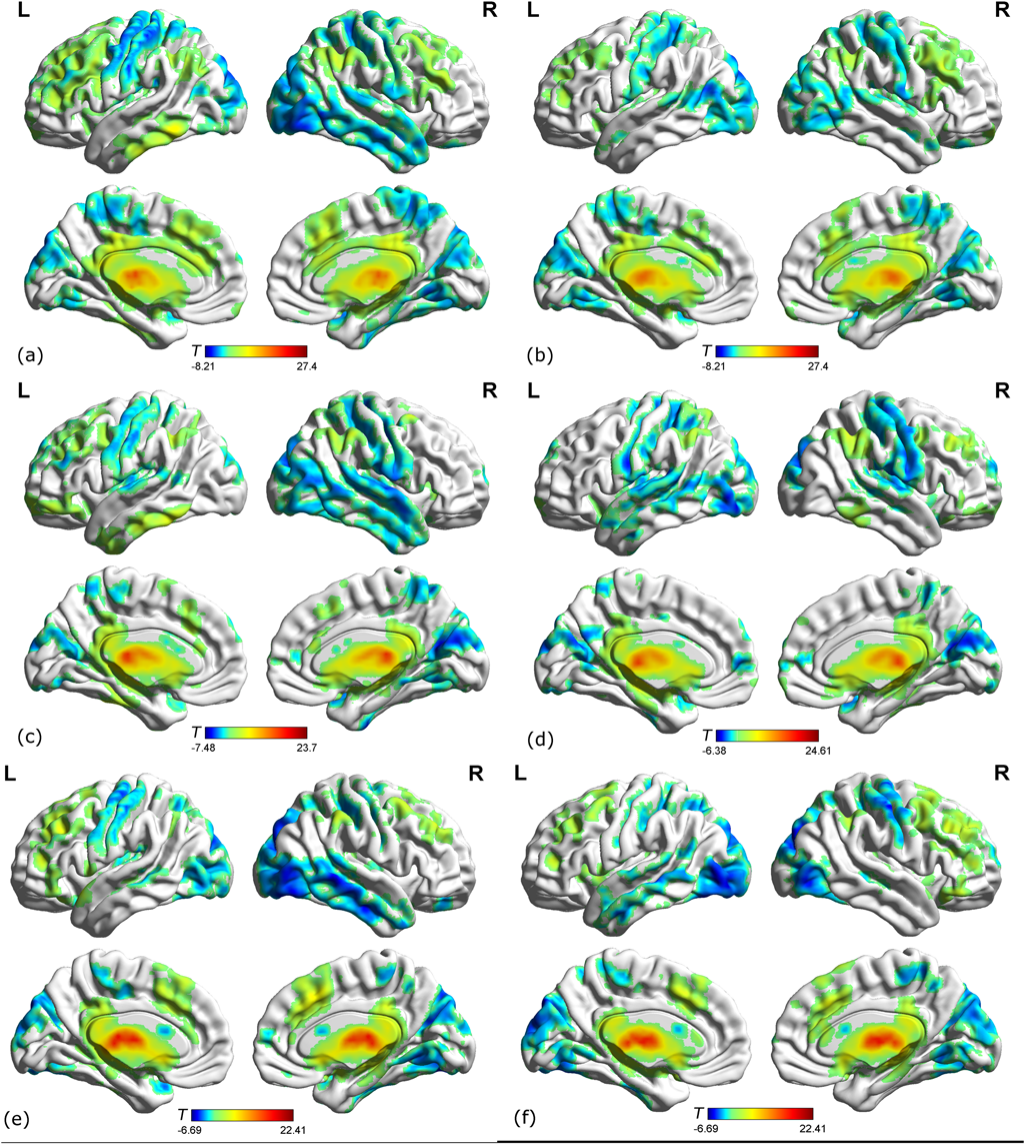
Brain regions show significant connectivity to the left/right thalamus in LMCI patients, EMCI patients and healthy age-matched control group (*P* < 0.01, 40 voxels, Alphasim corrected). (a) LMCI patients in the left thalamus; (b) LMCI patients in the right thalamus; (c) EMCI patients in the left thalamus; (d) EMCI patients in the right thalamus; (e) HC group in the left thalamus; (f) HC group in the right thalamus.

Fig. 1 shows the functional connectivity maps of healthy controls, EMCI and LMCI groups. Visual inspection indicated that the left and right thalami showed strong connectivity to a number of brain regions. Additionally, we also noted that these regions included the middle frontal gyrus (MFG), precentral gyrus (PreCG), postcentral gyrus (PoCG), supplementary motor area (SMA), middle temporal gyrus (MTG), superior temporal gyrus (STG), inferior parietal gyrus (IPG), anterior cingulate and paracingulate gyrus (ACG), and middle occipital gyrus (MOG) (Fig. 1a–f).

### 2. Group Differences in Functional Connectivity

**Fig 2:**
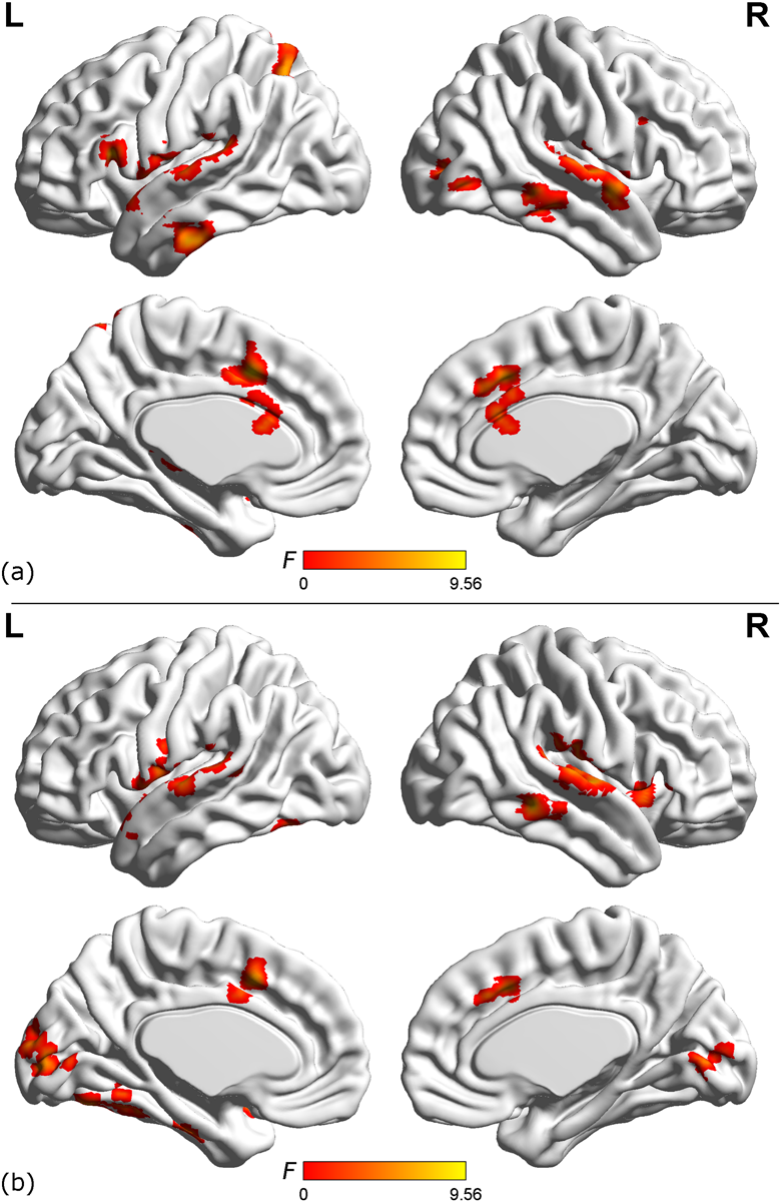
Brain areas with significant differences in the functional connectivity to the left thalamus (a) and right thalamus (b) among LMCI patients, EMCI patients and healthy control group (*P* < 0.01, 40 voxels, Alphasim corrected).

Fig. 2a shows the one-way ANOVA analysis of the left thalamus connectivity to all other brain regions among the three groups. Significant group differences in the left thalamus functional connectivity were observed in the IFG, medial frontal gyrus (mFG) and SMA, in addition to temporal regions such as the inferior temporal gyrus (ITG), MTG, STG, as well as the ACG, MOG and SPG. Fig. 2b shows the one-way ANOVA analysis of the right thalamus functional connectivity among the three groups. The main group differences were found in temporal regions such as the fusiform gyrus (FG), STG, and MTG. In addition, differences were found in the insula (INS), cuneus (CUN) and PreCG. See Table 3 for a detailed list of the regions.

**Table 3:** Regions showing functional connectivity differences in the left/right thalamus among the LMCI, EMCI and HC (*P* < 0.01, 40 voxels, corrected for multiple comparisons).

			MNI			
Brain regions	BA	Cluster size	x	y	z	Max ***Z***
**Functional connectivity to the left thalamus**						
L.ITG	20	28	-57	-15	-30	7.46
R.MTG	21	47	63	-36	-9	6.76
L.STG	38	13	-48	9	-12	6.25
R.MOG	18	22	36	-84	3	6.61
R.STG	22	72	57	0	-6	6.42
R.STG	41	24	39	-33	9	6.35
L.STG	42	73	-57	-33	15	7.45
L.IFG	45	41	-39	21	15	8.37
R.ACG	32	10	6	18	39	6.82
L.SMA/mFG	32	20	-6	12	42	8.94
L.SPG	7	46	-33	-63	54	7.39
**Functional connectivity to the right thalamus**						
L.FG	N/A	70	-33	-72	-15	6.97
L.STG	38	57	-45	12	-18	7.38
R.MTG	21	41	63	-39	-9	8.61
R.INS	13	20	45	12	3	5.65
L.CUN	17	9	-12	-90	3	7.2
L.PreCG	6	25	-57	-3	12	4.93
L.STG	42	64	-63	-27	12	6.81
R.STG	22	109	60	-12	3	7.58

Abbreviations: ITG = inferior temporal gyrus; MTG = middle temporal gyrus; STG = superior temporal gyrus; MOG = middle occipital gyrus; IFG = inferior frontal gyrus; ACG = anterior cingulate and paracingulate gyrus; SMA = supplementary motor area; mFG = medial frontal gyrus; SPG = superior parietal gyrus; FG = fusiform gyrus; INS = insula; CUN = cuneus; PreCG = precentral gyrus; R = right side; L = left side; BA = Brodmann’s area; N/A: not applicable.

**Fig 3:**
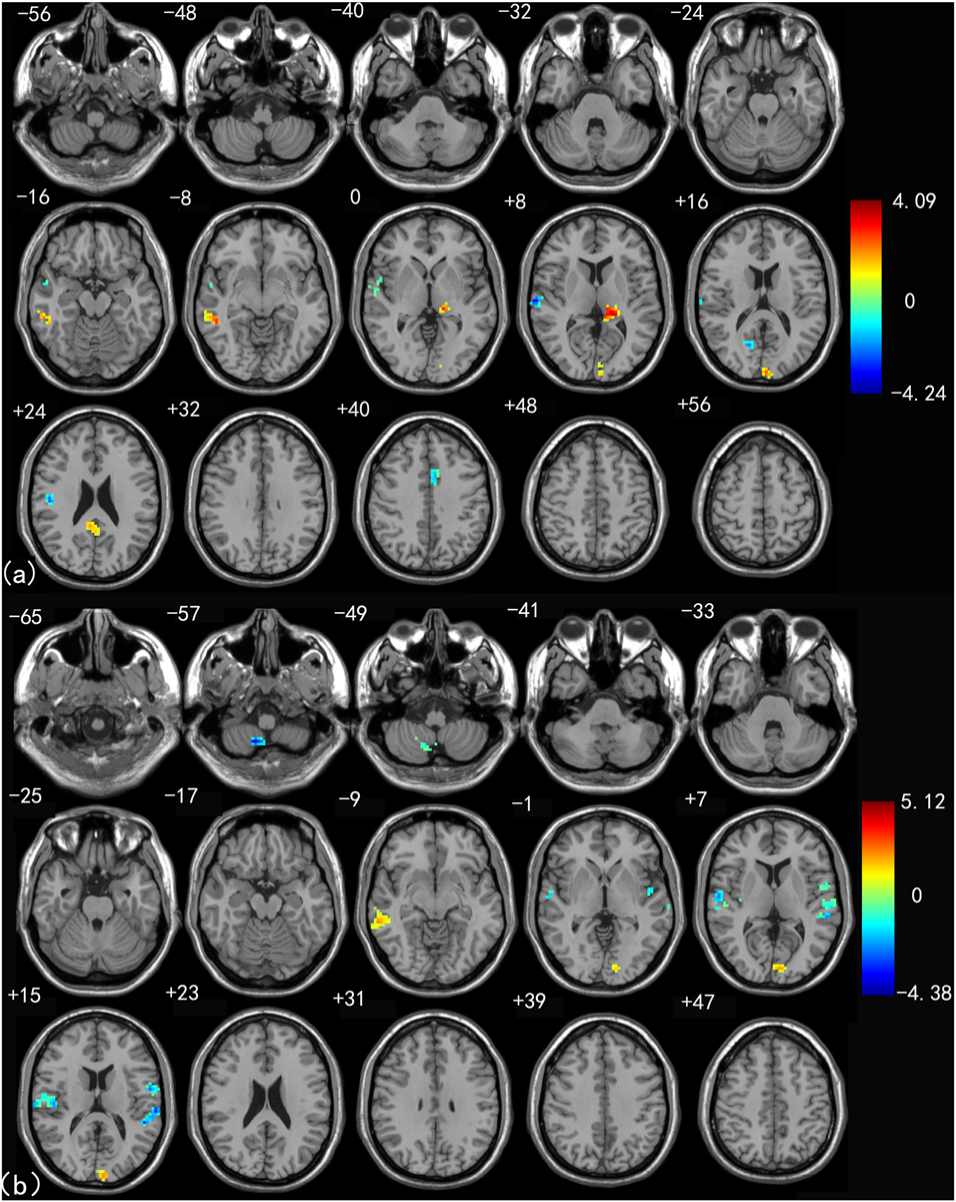
Left (a) and right (b) thalamus connectivity difference maps between the EMCI patients and healthy control group (*P* < 0.01, 40 voxels, Alphasim corrected).

Fig. 3 shows thalamic functional connectivity differences between EMCI and healthy controls. Compared to healthy controls, the EMCI group showed prominent increased connectivity between the thalamus and a number of other brain areas. Increased connectivity of the left thalamus was observed in the regions of the left ITG, left FG, right MOG, right MTG, and right precuneus (PCu). Decreased connectivity of the left thalamus was seen in both the left and right STG and left mFG extending into SMA (Fig. 3a). Significant increased connectivity of the right thalamus was found in the regions of the left FG and right MTG. Interestingly, decreased connectivity of the right thalamus was similar with connectivity of the left thalamus (Fig. 3b). See Table 4 for a detailed list of these regions.

**Table 4:** Regions showing functional connectivity differences of the left/right thalamus between the EMCI and HC group (*P* < 0.01, 40 voxels, corrected for multiple comparisons).

			MNI				HC	EMCI			
Brain regions	BA	Cluster size	x	y	z	Max ***Z***	*T*	*T*		*CC*	*P*
**Functional connectivity of the left thalamus**											
**EMCI < HC**											
L.STG	38	27	-51	12	-12	-3.47	5.891	3.095	**↓+**	0.095	0.486
R.STG	41	65	45	-33	12	-3.26	6.124	3.671	**↓+**	**0.502**	**0.009**
L.mFG/SMA	32	60	-6	12	42	-3.67	-1.352	-4.366	**↓-**	0.081	0.553
**EMCI > HC**											
L.ITG	20	35	-57	-15	-30	3.32	-4.353	-0.695	**↑-**	0.302	0.099
L.FG	N/A	47	-33	-69	-15	3.53	1.608	4.332	**↑+**	0.106	0.499
R.MTG	20	61	51	-42	-12	3.68	-5.226	-2.113	**↑-**	0.163	0.772
R.MOG	18	37	36	-84	3	3.86	2.603	6.752	**↑+**	0.039	0.771
R.PCu	7	18	9	-78	45	3.28	-5.613	-1.166	**↑-**	0.094	0.821
**Functional connectivity of the right thalamus**											
**EMCI < HC**											
L.STG	38	96	-45	12	-18	-3.86	5.591	2.575	**↓+**	0.148	0.866
R.STG	22	154	60	-12	3	-3.89	6.422	3.564	**↓+**	0.152	0.896
L.mFG/SMA	6	35	-3	12	45	-3.69	-0.966	-4.129	**↓-**	0.096	0.45
**EMCI>HC**											
L.FG	N/A	125	-33	-69	-15	3.92	2.033	5.172	**↑+**	0.206	0.109
R.MTG	21	53	63	-39	-9	3.30	-4.223	-2.124	**↑-**	0.306	0.097

Abbreviations: STG = superior temporal gyrus; mFG = medial frontal gyrus; SMA = supplementary motor area; ITG = inferior temporal gyrus; FG = fusiform gyrus; MTG = middle temporal gyrus; MOG = middle occipital gyrus; PCu = precuneus; R = right side; L = left side; BA = Brodmann’s area; BA = Brodmann’s area; N/A = not applicable.

***T***: functional connectivity strength of the left/right thalamus and *T* value was obtained by a one sample t-test; ***CC***: correlation coefficient; **“↓+":** decrease in positive functional connectivity; **”↓-“**: decrease in negative functional connectivity; **”↑+“**: increase in positive connectivity; **”**

**↑-“**: increase in negative connectivity;”

The last two columns show the correlation coefficient and corresponding *p* value between the strength of functional connectivity and MMSE scores, and the results for a threshold of ***p* < 0.05** are shown in bold.

**Fig 4:**
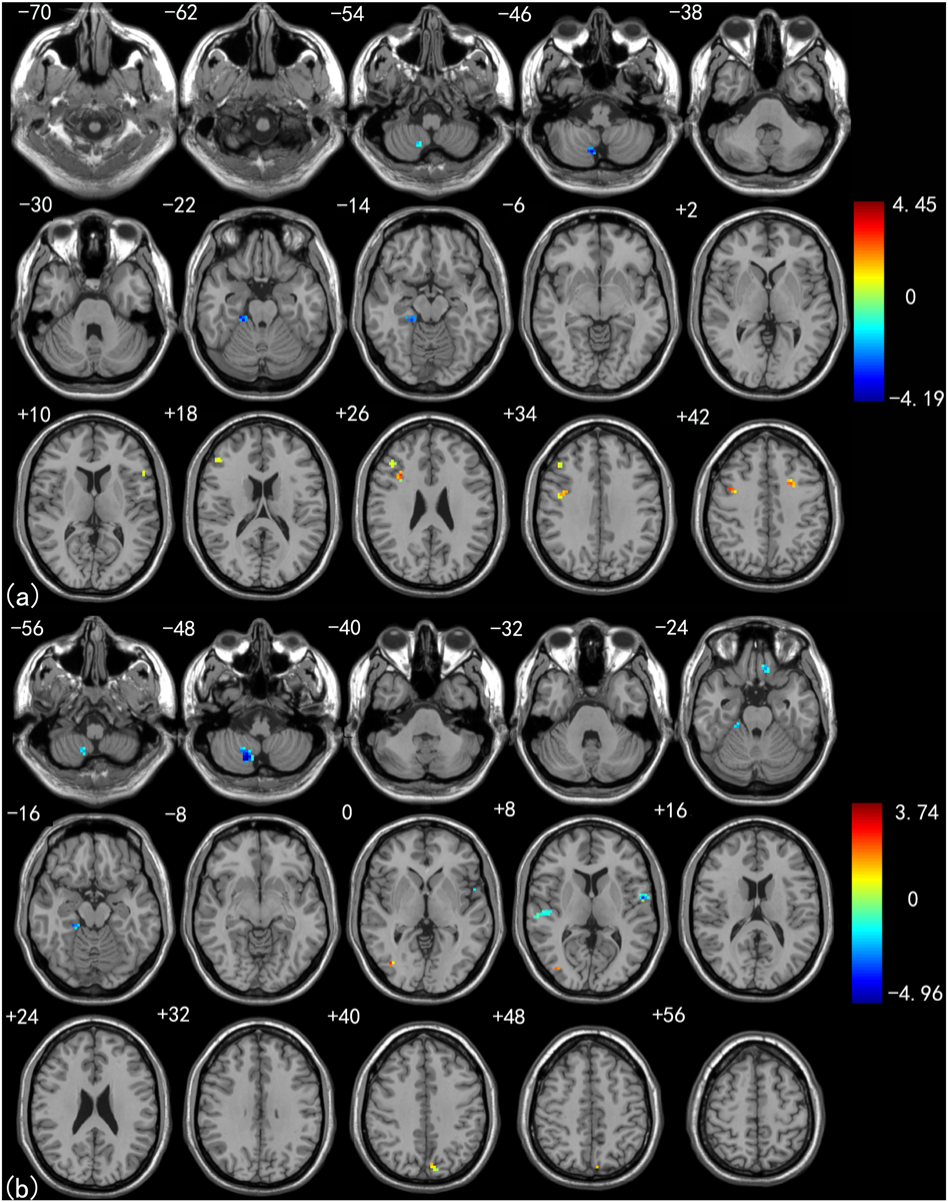
Left (a) and right (b) thalamus connectivity difference maps between LMCI patients and healthy control group (*P* < 0.01, 40 voxels, Alphasim corrected).

Fig. 4 shows thalamic functional connectivity differences between LMCI and healthy controls. Significant increased connectivity of the left thalamus to other brain regions was mainly located in the left FG, left ITG, left MTG, and left PCu. In addition, decreased connectivity in the left thalamus was found in the right FG, right STG and left SMA (Fig. 4a). As for the right thalamus, increased connectivity of the LMCI was found in the regions including the left FG and left PCu, while decreased connectivity was seen in the right STG, right INS, right FG, left MTG and left MOG (Fig. 4b). See Table 5 for a detailed list of these regions.

**Table 5:** Regions showing functional connectivity differences of the left/right thalamus between the LMCI and HC group (*P* < 0.01, 40 voxels, corrected for multiple comparisons).

			MNI				HC	LMCI			
Brain regions	BA	Cluster size	x	y	z	Max ***Z***	*T*	*T*		*CC*	*P*
**Functional connectivity of the left thalamus**											
**LMCI < HC**											
R.FG	N/A	12	21	-33	-18	-3.86	4.327	2.332	**↓+**	0.088	0.492
R.STG	20	27	45	-6	-18	-3.05	6.889	1.553	**↓+**	0.094	0.452
L.SMA	6	28	0	6	54	-3.07	-1.368	-4.257	**↓-**	0.118	0.460
**LMCI > HC**											
L.FG	20	26	-60	-21	-30	3.63	0.994	4.357	**↑+**	0.105	0.511
L.ITG/MTG	20	29	-63	-48	-12	3.13	-5.341	-2.641	**↑-**	**0.412**	**0.010**
L.PCu	7	34	-6	-78	42	2.84	-4.225	-1.002	**↑-**	**0.506**	**0.009**
**Functional connectivity of the right thalamus**											
**LMCI < HC**											
R.STG/INS	38	70	36	21	-30	-3.99	6.227	2.651	**↓+**	0.203	0.098
R.FG	N/A	17	21	-33	-18	-3.98	4.894	2.338	**↓+**	0.069	0.865
L.MTG/MOG	39	33	-45	-69	15	-3.14	-0.851	-4.159	**↓-**	0.105	0.512
**LMCI > HC**											
L.FG	20	37	-33	-18	-30	3.36	3.004	5.213	**↑+**	0.097	0.453
L.PCu	7	30	-6	-78	42	2.99	-6.225	-2.316	**↑-**	**0.396**	**0.015**

Abbreviations: FG = fusiform gyrus; STG = superior temporal gyrus; SMA = supplementary motor area; ITG = inferior temporal gyrus; MTG = middle temporal gyrus; PCu = precuneus; R = right side; L = left side; BA = Brodmann’s area; N/A = not applicable.

***T***: functional connectivity strength of the left/right thalamus and *T* value was obtained by a one sample t-test; ***CC***: correlation coefficient; **“↓+":** decrease in positive functional connectivity; **“↓-”**: decrease in negative functional connectivity; **“↑+”**: increase in positive connectivity; **“↑-”**: increase in negative connectivity; “

The last two columns show the correlation coefficient and corresponding *p* value between the strength of functional connectivity and MMSE scores, and the results for a threshold of ***p* < 0.05** are shown in bold.

**Fig 5:**
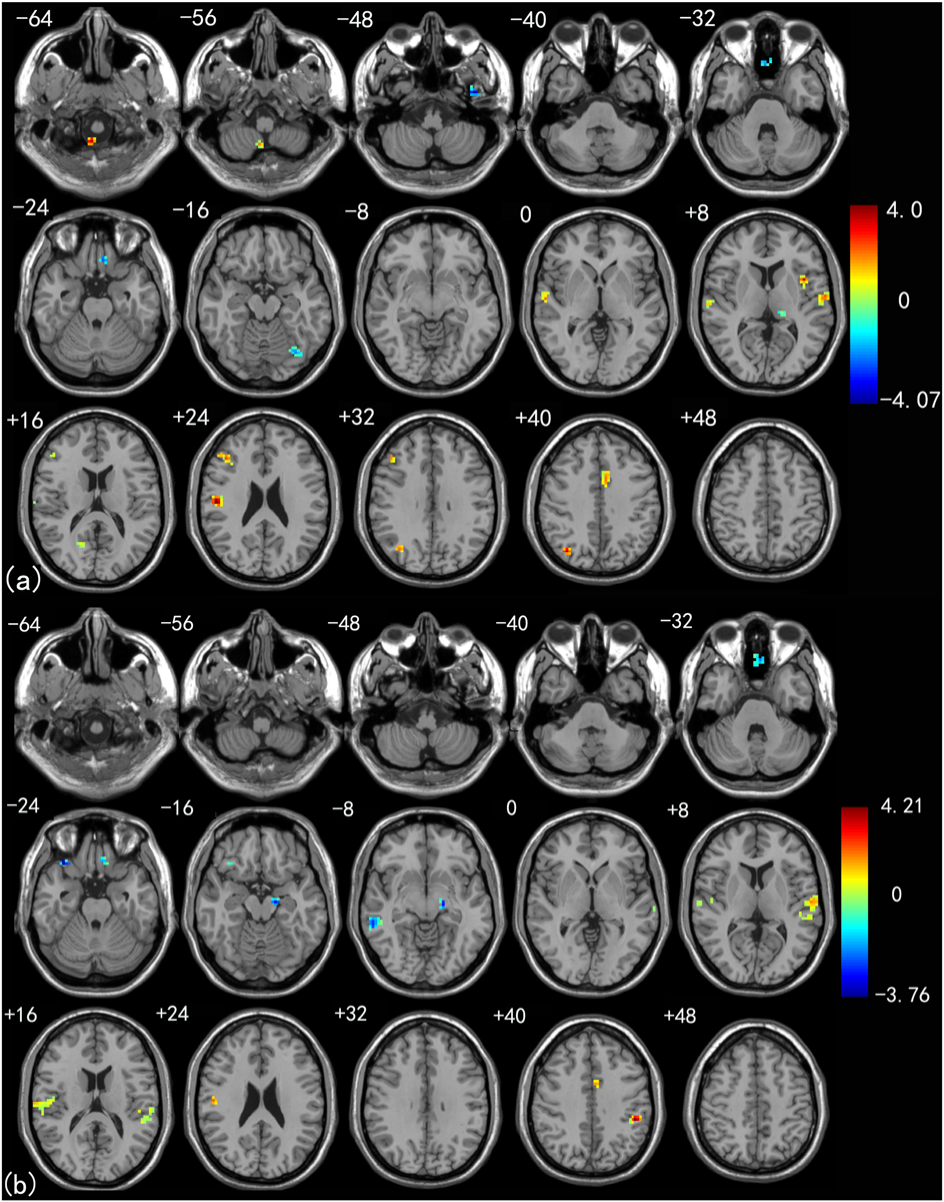
Left (a) and right (b) thalamus connectivity difference maps between LMCI patients and EMCI patients (*P* < 0.01, 40 voxels, Alphasim corrected).

Fig. 5 shows a direct comparison of functional connectivity differences between the LMCI and EMCI groups. Compared with the EMCI subjects, the LMCI subjects showed significant increased functional connectivity with the left thalamus in the left STG, right PoCG and right MFG. Decreased functional connectivity with the left thalamus was found in the right FG, left MFG, right thalamus (THA), and right PreCG (Fig. 5a). As for the right thalamus, the LMCI subjects showed increased functional connectivity in the left MTG, right PoCG, left STG and right MFG. In addition, decreased connectivity of the right thalamus was seen in the bilateral FG, right MTG, and left hippocampus (HIP) (Fig. 5b). See Table 6 for a detailed list of these regions.

**Table 6:** Regions showing functional connectivity differences of the left/right thalamus between the LMCI and EMCI group (*P* < 0.01, 40 voxels, corrected for multiple comparisons).

			MNI				EMCI	LMCI			
Brain regions	BA	Cluster size	x	y	z	Max ***Z***	*T*	*T*		*CC*	*P*
**Functional connectivity of the left thalamus**											
**LMCI< EMCI**											
R.FG	N/A	34	36	-42	-18	-2.87	3.997	1.234	**↓+**	0.068	0.701
L.MFG	11	42	-42	51	-12	-3.58	-2.304	-5.159	**↓-**	0.145	0.471
R.THA	N/A	15	3	-24	12	-4.07	4.552	1.364	**↓+**	0.120	0.511
R.PreCG	N/A	21	18	-18	75	-3.19	-3.001	-6.283	**↓-**	-0.101	0.620
**LMCI> EMCI**											
L.STG	42	56	-54	-33	15	3.09	2.558	5.309	**↑+**	-0.262	0.188
R.PoCG	3	35	54	-18	24	3.18	-4.912	-1.399	**↑-**	**0.381**	**0.014**
R.MFG	8	49	45	30	39	3.06	-6.003	-2.364	**↑-**	-0.233	0.243
**Functional connectivity of the right thalamus**											
**LMCI< EMCI**											
L.FG	37	47	-36	-57	-12	-3.08	5.113	1.958	**↓+**	0.124	0.532
R.FG	N/A	40	30	-36	-24	-3.11	4.995	1.309	**↓+**	0.145	0.476
R.MTG	21	80	63	-39	-9	-3.52	-0.947	-4.016	**↓-**	**0.413**	**0.010**
L.HIP	35	16	-18	-18	-9	-3.61	-3.225	-6.301	**↓-**	-0.305	0.077
**LMCI> EMCI**											
L.MTG	20	17	-45	-21	-12	3.58	-3.965	-1.792	**↑-**	0.182	0.154
R.PoCG	43	52	57	-18	21	2.94	-5.631	-1.992	**↑-**	-0.177	0.180
L.STG	22	51	-66	-12	3	3.02	0.967	3.152	**↑+**	0.008	0.983
R.MFG	9	42	48	30	39	3.13	-4.696	-1.564	**↑-**	0.221	0.256

Abbreviations: FG = fusiform gyrus; MFG = middle fusiform gyrus; THA = thalamus; PreCG = precentral gyrus; STG = superior temporal gyrus; PoCG = postcentral gyrus; MTG = middle temporal gyrus; HIP = hippocampus; R = right side; L = left side; BA = Brodmann’s area; N/A = not applicable.

***T***: functional connectivity strength of the left/right thalamus and *T* value was obtained by a one sample t-test; ***CC***: correlation coefficient; **“↓+":** decrease in positive functional connectivity; **“↓-”**: decrease in negative functional connectivity; **“↑+”**: increase in positive connectivity; **“↑-”**: increase in negative connectivity; “

The last two columns show the correlation coefficient and corresponding *p* value between the strength of functional connectivity and MMSE scores, and the results for a threshold of ***p* < 0.05** are shown in bold.

Each second column of tables 4–6 shows the detailed condition about decreased and increased connectivity for the identified regions. For example, the *t* value between the left precuneus and right thalamus in the LMCI group was-2.316, but in HC group it was-6.225. We can understand that the left precuneus had increased negative functional connectivity (decrease in the absolute value) when comparing LMCI with the HC group Similar explanations apply to the other regions we identified

### 3. Relationship between Functional Connectivity Values and Clinical Variables

To identify the relationship between the strength of functional connectivity and the clinical scores of LMCI and EMCI, the average strength of functional connectivity of all voxels in the above regions was extracted separately. We detected several regions which had significant positive correlations between functional connectivity values and MMSE scores. These brain regions included the right STG, left ITG/MTG, left PCu, right PoCG and right MTG. No significant correlations were found between functional connectivity values and MMSE scores in other above-mentioned brain regions (Tables 4–6).

## Discussion

To reveal the common and distinct pathophysiology of aMCI subtypes, we compared the functional connectivity of thalamus in patients and controls. Our research investigated the left and right thalamus functional connectivity with the other brain regions in spontaneous brain activity by measuring resting-state fMRI signals. We found that there were significant differences in the thalamic functional connectivity among the LMCI, EMCI and healthy controls. Moreover, the brain regions that showed significant differences in the post-hoc two-sample *t*-test between EMCI and HC were different from those areas between LMCI and HC. These findings supported our hypothesis to a great extent and provided a new sight to understand the two important states of aMCI.

### 1. Thalamus Functional Connectivity Differences between EMCI and HC

In the present work, we found that both the left and right thalami showed decreased functional connectivity with the bilateral STG, left mFG and left SMA. The STG is mainly involved in auditory processing and language reception. Functional studies of the STG in animals and in humans using electrophysiology and PET/fMRI emphasize the STG’s role as part of a cortical network in the interpretation, production and self-monitoring of language. In schizophrenic patients, functional studies of this region were abnormal especially when patients performed language tasks or experienced hallucinations [35]. The decreased connectivity between the left/right thalamus and STG in EMCI implied that the interpretation and self-monitoring of the language function was impaired. More importantly, the strength of functional connectivity between the thalamus and STG was positively correlated with MMSE, which indicated that cognitive ability was significantly correlated with the functional connectivity index of this region. The other region of the mFG is located in the default mode network (DMN), a network functionally connected with the PCC/PreCU, mFG and IPL regions [36–38]. Degeneration of mFG connectivity with the left and right thalami may contribute to the disorders of working memory, attention and executive function in EMCI patients. This finding may also provide further evidence that disrupted thalamo-default mode network functional connectivity patterns underlie the impaired cognitive ability of aMCI patients. The decreased connectivity with the left/right STG and left mFG is consistent with recent research by Hughes et al [15], who found a significant reduction in the volume of the whole thalamo-cortical unit such as the temporal and frontal region projections with increasing age. We also observed decreased connectivity between the left SMA and left/right thalami. This implied that the alteration of connectivity has been extended to the primary motor cortex in EMCI.

As for the increased connectivity, we found both the left and right thalamus showed increased functional connectivity with the left FG and right MTG, and the left thalamus showed increased connectivity with the left ITG, right MOG and right PCu. The different patterns in the left and right thalamus may be due to the different functions of different sides in the human brain. According to previous research, FG is considered as a significant brain region in the processing of memory. Functional imaging studies have demonstrated that the FG usually showed increased activity in MCI during cognitive tasks, even during the resting-state [39–41]. The ITG is the end visual processing area of the ventral visual pathway and is related to visual working memory [42]. The MTG is connected with verbal and visual semantic knowledge [43] and is also related to verbal short-term memory [44]. A previous study showed significant compensatory increased activation of the MTG in early stages of AD patients during a working memory task, which demonstrated that access to semantic knowledge was preserved [45]. Thus, our finding of increased connectivity between the thalamus and temporal lobe may make known a compensatory reallocation of functional connectivity. Panayiotopoulos et al.[46]’s study showed that visual hallucinations were relevant to the MOG. The symptom of visual hallucinations in Alzheimer’s disease may be attributed to the pathology of functional connectivity between the MOG and the left thalamus. However, the pathogenesis of visual hallucinations still unclear, we will carefully explore it in the future.

Wang et al. [22] showed that the PCu presented decreased connectivity in the right thalamus in MCI, while our results showed increased connectivity. This outcome might due to choosing different MCI subsets and further research be needed to explain this discrepancyapp:addword:discrepancy. The thalamus pulvinar has widespread connectivity with the posterior parietal lobe and the PCu, which are pathological biomarkers in AD patients [47]. Just as noted in the discussion above, the PCu is also a part of the default mode network. Several research studies employed resting-state fMRI and graph theory approaches to systematically investigate the topological organization of the functional connectome in AD and/or MCI, and found that altered brain regions were mainly located in the default mode network and the temporal lobe [48, 49]. The disruption of these circuits in subsets of the aMCI suggested cognitive changes. Some of the cognitive symptoms in aMCI, such as visual-spatial perception syndrome and visual hallucinations, may be due to the pathology of the thalamus and thalamus-related networks.

### 2. Thalamic Functional Connectivity Differences between LMCI and HC

We observed decreased connectivity of the thalamus in the right STG and left SMA, as well as increased connectivity of the thalamus in the left FG, left ITG/MTG and left PCu. The decreased and increased functional connectivity with the thalamus in the above six regions have been discussed in the previous section. We also found that functional connectivity values between the thalamus and several regions including the left ITG/MTG and left PCu were significantly correlated with MMSE (Table 5). Taken together, our data suggested that aMCI patients could recruit other brain regions involved in working memory and use different cognitive mechanisms to compensate for impaired memory. Noticeably, the six regions were also presented in the EMCI group (Table 4). This finding suggested that the lesions and compensation may initially start from these regions.

Note that decreased functional connectivity of the left thalamus was found in the right FG and increased connectivity of the left thalamus was found within the left FG. This phenomenon may be surmised to be a new compensation mechanism that recruited the other side of the brain region to maintain basic physiological function when the one side of the region was impaired. What’s more, the increased functional connectivity between the left FG and the left thalamus was consistent with one of our studies about the functional connectivity of the fusiform (Cai *et al*., submitted for publication). Similarly, the decreased functional connectivity between the left MTG and the right thalamus and the increased connectivity between the left MTG and the left thalamus were considered as the same compensation mechanism. However we have not found hard evidence to support this viewpoint, which also needs to be explored in our future research.

Besides, we detected some other regions that were not found in the EMCI group including the right INS and left MOG (decreased connectivity with the right thalamus), as well as the left PCu (increased connectivity with the left thalamus). The INS has diverse functions, including taste, language, auditory processing, visceral sensorimotor response, somatic sensation, and movement. According to previous research, the insula was strongly coactivated with both the putamen and caudate. A combined transcranial magnetic stimulation (TMS) and fMRI study also uncovered the functional connectivity between the insula and thalamus [50]. The decreased connectivity between the right thalamus and right INS indicated that the pathway was impaired between them and further impacted normal functions such as auditory processing, somatic sensation and movement in LMCI patients. Our finding provided new evidence that functional connectivity between the INS and thalamus is of considerable importance [7]. The MOG is located in the primary visual cortex. Recently, Zou and colleagues investigated the functional relationship between the thalamus and visual cortex using resting state fMRI and provided evidence for connectivity of the MOG [51]. Our findings indicated that damaged thalamo-visual network may cause impairment of visual function in aMCI. Interestingly, the connectivity between the thalamus and the MOG was increased in the EMCI group, whereas the connectivity between the thalamus and the MOG was decreased in the LMCI group. This finding suggested that the compensatory function of the MOG degenerated into pathological change as the disease progressed.

### 3. Thalamus Connectivity Differences between LMCI and EMCI

We observed connectivity differences between LMCI and EMCI in regions including the frontal lobe regions (MFG, PreCG, PoCG), temporal lobe regions (STG, MTG, FG, HIP), and thalamus. Noticeably, the strength of functional connectivity between the thalamus and the two identified brain regions including PoCG (increased connectivity) and MTG (decreased connectivity) was positively correlated with the MMSE. In addition, the left FG and left MTG in LMCI had stronger increased functional connectivity than that in the EMCI group. The left MFG had stronger decreased functional connectivity than that in the EMCI group. Based on the evidence we found and the discussion of these regions in the previous section, this finding implied that the LMCI have more memory impairment and compensation requirement compared to EMCI patients and it also conform to the degradation and progress of the disease.

The HIP is the primary site of neuronal degeneration in AD [52] and Zarei et al. [16] combined shape and connectivity to successfully detect regional thalamus atrophy in AD. The decreased connectivity between the thalamus and HIP may be ascribed to degeneration alterations in the thalamo-hippocampal network. Pergola et al [53] found that the thalamus is a vital region for recognition accompanied by recall, where the activity of the thalamo-temporal network selectively predicts better memory performances during retrieval across subjects and this confirms the foremost role of this network in recall and recollection. Besides, our findings of the changed regions in the frontal lobe, temporal lobe and hippocampus were consistent with Zarei et al. [16]’s study showing that the regional thalamus atrophy in AD was mainly connected with the frontal cortex, temporal cortex and hippocampus.

Additionally, we observed that the LMCI showed decreased left thalamus to right thalamus connectivity compared with the EMCI. As shown in previous research [16, 53], decreased functional connectivity between the left and right thalamus in LMCI may be due to the regional thalamus atrophy, because atrophy can lead to the loss of thalamus function. In summary, as the stage moved closer to AD, the LMCI showed more abnormal functional connectivity of the thalamus.

### 4. Limitations

There were several limitations in our research. First of all, we selected the data from the database ADNI and no structural MR images were provided publicly. Consequently, in the method part of image preprocessing, we spatially normalized images not to the structural images of the subjects but to the standard EPI functional template which is a built-in template in SPM. Although the normalized method can produce a good result, it is still not the best, which is our main concern. Recently, some fMRI investigators have suggested that functional results could potentially be influenced by structural differences between groups [54, 55]. Future studies that combine functional connectivity with structural connectivity techniques (e.g., diffusion tensor imaging (DTI) with probabilistic tractography) will be helpful in researching the relationship between abnormal functional connectivity and structural abnormalities in aMCI patients. Secondly, the thalamus is a complex brain area that can be divided into several different subdivisions according to the historical cytoarchitectonic atlas [56, 57]. We should investigate the functional connectivity in each subdivided area of the thalamus to precisely illustrate the complex functional deficits of the subsets of aMCI. In the future, we will explore a standard template for sub-dividing the thalamus and study the connectivity of each subdivided areas of the thalamus in resting state fMRI. Thirdly, In our study, the global signal is used as a regressor to remove the associated variance in fMRI analyses [58]. However, there is much debate about regressing global mean signal. Further study on the comparison between regressing global mean signal and without regressing global mean signal is needed.

In summary, the present work investigated functional connectivity of the thalamus with all the other brain regions among the three groups. We found significant difference between each pair groups. These brain regions that showed significant differences were mainly located in the thalamo-related networks including thalamo-hippocampus, thalamo-temporal, thalamo-visual, and thalamo-default mode network. The decreased functional connectivity of the thalamus might suggest reduced functional integrity of thalamo-related networks and increased functional connectivity indicated that aMCI patients could use additional brain resources to compensate for the loss of cognitive function. Our study provided a new sight to understand the two important states of aMCI and revealed resting-state fMRI is an appropriate method for exploring pathophysiological changes in aMCI.

## Supporting Information

S1 Fig There are six normalized maps (a)-(f) to prove that the EPI template can give a good result. Note: The EPI template is a built-in functional template in SPM (http://www.fil.ion.ucl.ac.uk/spm/)
(TIF)
