# Supplementary figures and images for: Changes in Thalamic Connectivity in the Early and Late Stages of Amnestic Mild Cognitive Impairment: A Resting-State Functional Magnetic Resonance Study from ADNI
Source: PLoS One. 2015 Feb 13;10(2):e0115573. doi: 10.1371/journal.pone.0115573 (PMC4332494; doi:10.1371/journal.pone.0115573)

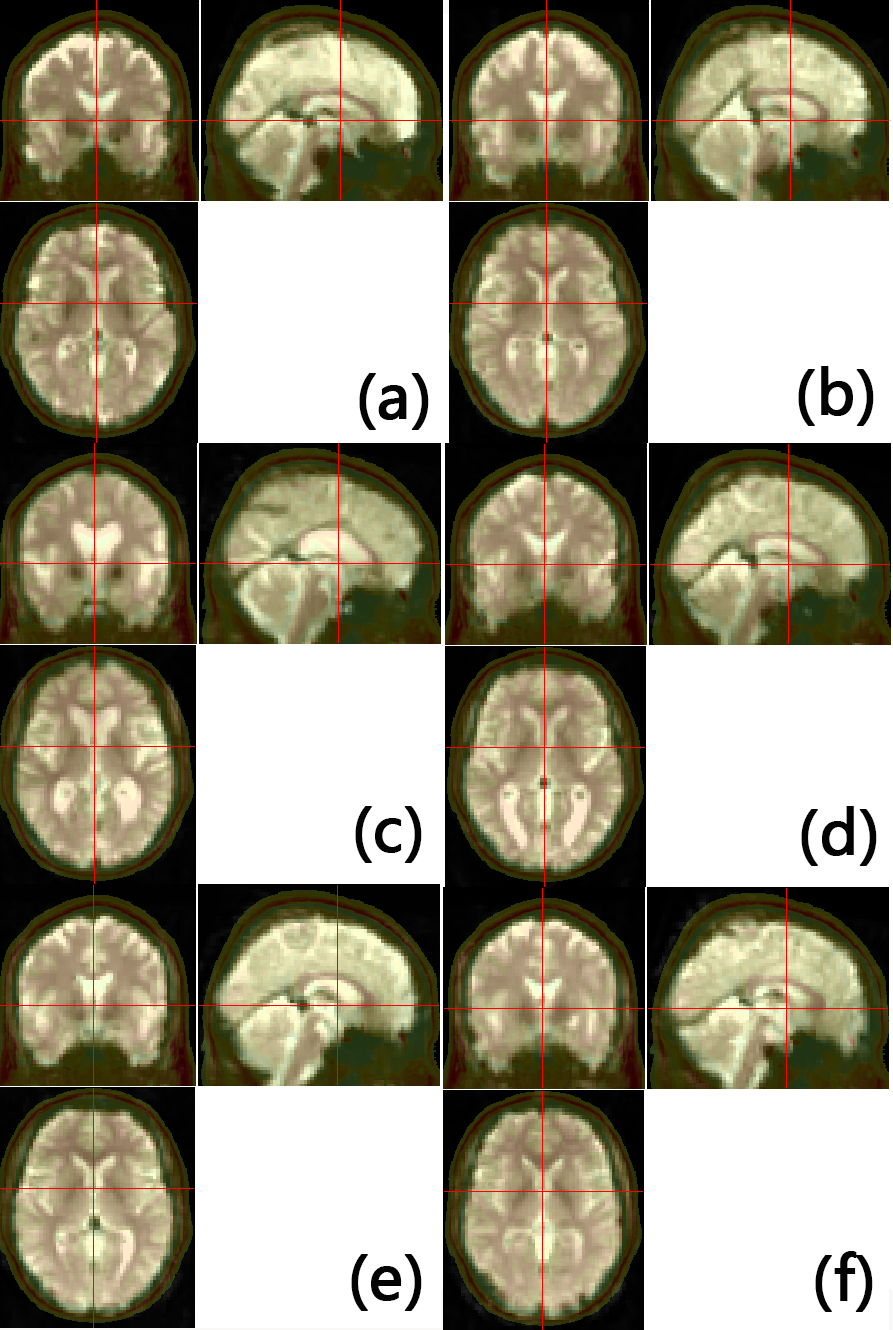

Supplement: S1 Fig — Note: The EPI template is a built-in functional template in SPM (http://www.fil.ion.ucl.ac.uk/spm/) (TIF) [file pone.0115573.s001.tif]
